# From Conventional to Next Generation Sequencing of Epstein-Barr Virus Genomes

**DOI:** 10.3390/v8030060

**Published:** 2016-02-24

**Authors:** Hin Kwok, Alan Kwok Shing Chiang

**Affiliations:** Department of Paediatrics and Adolescent Medicine, Li Ka Shing Faculty of Medicine, The University of Hong Kong, Hong Kong, China; hinkwok@hku.hk

**Keywords:** Epstein-Barr virus, Next-generation sequencing, target capture, genome assembly

## Abstract

Genomic sequences of Epstein–Barr virus (EBV) have been of interest because the virus is associated with cancers, such as nasopharyngeal carcinoma, and conditions such as infectious mononucleosis. The progress of whole-genome EBV sequencing has been limited by the inefficiency and cost of the first-generation sequencing technology. With the advancement of next-generation sequencing (NGS) and target enrichment strategies, increasing number of EBV genomes has been published. These genomes were sequenced using different approaches, either with or without EBV DNA enrichment. This review provides an overview of the EBV genomes published to date, and a description of the sequencing technology and bioinformatic analyses employed in generating these sequences. We further explored ways through which the quality of sequencing data can be improved, such as using DNA oligos for capture hybridization, and longer insert size and read length in the sequencing runs. These advances will enable large-scale genomic sequencing of EBV which will facilitate a better understanding of the genetic variations of EBV in different geographic regions and discovery of potentially pathogenic variants in specific diseases.

## 1. Introduction

Epstein–Barr virus is a gamma-herpesvirus which infects more than 90% of the world’s population. It is associated with cancers such as Hodgkin’s lymphoma, Burkitt’s lymphoma, gastric cancer, nasopharyngeal carcinoma, and other conditions, such as post-transplant lymphoproliferative diseases and infectious mononucleosis. The geographical distribution of EBV strains and the endemic incidence of EBV-associated diseases have prompted investigations of whether there are distinct strains of EBV that contribute to the disease process. EBV strains have previously been characterized in NPC and other disease at various loci, including EBER-1 and -2, LMP1, BHRF1, BZLF1, and EBNA1 in samples from China, South Asia, and Northern Africa [1,2,3,4,5,6]. However, these early studies have only been focused on the individual viral genes. Very few studies have been carried out to investigate how genetic variations of EBV on a whole-genome scale might influence infection or pathogenesis of EBV-associated diseases. The large genome size of EBV relative to other viruses renders large-scale sequencing of EBV genomes cost- and time-prohibitive. Advances in next-generation sequencing technology have promoted the determination of EBV genomic sequences and reignited the interest of studying the viral genome and its association to diseases. This review provides an overview of how the sequences of published EBV genomes have been determined, and the bioinformatics analyses involved.

## 2. Conventional Shotgun Sequencing of EBV Genomes

Conventional shotgun sequencing involved the techniques of digestion of genomic DNA with restriction enzymes, cloning, and dideoxynucleotide (Sanger) sequencing. It has been widely applied to genomic sequencing of organisms, such as humans and Epstein-Barr viruses.

The prototypical strain B95-8 was the first complete EBV genome sequenced. An 833L cell line was obtained by culturing lymphocytes from an individual with infectious mononucleosis (IM), then the EBV released from the 883L line was used to infect marmoset B cells, resulting in the B95-8 line [7]. EcoRI- and BamHI-digested fragments have first been cloned and restriction maps of these clones were obtained [8,9,10]. The DNA sequence was analyzed by constructing M13 subclone libraries, followed by random sequencing using the dideoxynucleotide method [11]. Due to its early availability, B95.8 has been extensively mapped for transcripts, promoters, open reading frames, and other structural elements, by means of Northern blotting and other methods [12,13]. By filling in the 11 kb deletion in B95-8 with Raji EBV sequence, this chimeric EBV genome serves as the type 1 EBV reference sequence [14] and is widely used in genomic analysis in later studies.

GD1 (Guangdong strain 1) was an EBV genome derived from NPC patients from the Guangdong province of Southern China. It was isolated by infecting umbilical cord mononuclear cells by EBV from the saliva of the patient [15]. The EBV DNA was PCR amplified and sub-cloned, then sequenced by conventional Sanger sequencing. More than 1400 point mutations were identified by comparing against the type 1 reference, some of which were also found in high frequency in NPC biopsies of the same geographical region, hence, suggesting that GD1 is representative of the EBV strains found in Southern Chinese NPC patients.

AG876 was originated from a Ghanaian case of Burkitt's lymphoma and was the first complete type 2 EBV genome published [16]. Sequence analysis was performed by first constructing cosmid libraries through Sau3AI digestion, followed by dideoxynucleotide sequencing. The determination of AG876 sequence has made comparison of whole-genome comparison of type 1 and 2 EBV possible. It had validated that the two major types of EBV are generally very similar outside the divergent regions at EBNA2 and EBNA3 genes.

B95-8, GD1, and AG876 are the products of immense effort and they represent EBVs of different geographical origins (B95-8 as an American strain, GD1 as a Southern Chinese strain, and AG876 as an African strain) and different diseases (B95-8 from IM, GD1 from NPC, and AG876 from Burkitt’s lymphoma). A summary of sequencing methods of these three and other published EBV genomes are shown in Table 1.

## 3. Next-Generation Sequencing of EBV Genomes

### 3.1. Direct Next-Generation Sequencing without EBV Enrichment

GD2, as with GD1, was an EBV genome from NPC patient of Guangdong province. However, instead of capturing the EBV from saliva of the patient, GD2 was directly sequenced from an NPC tumor [17]. Its genome was obtained as a small subset of sequence data from next-generation sequencing of total DNA sequences derived from the primary NPC tumor, hence, can be acknowledged as the first natural EBV genome determined by next-generation sequencing technology [17]. This study illustrated that it is possible to directly determine EBV genomes in tumor biopsies without enrichment, though only reads 0.0141% of the total reads were mapped to EBV and used to construct GD2 [17].

C666-1 is a sub-clone of C666, an epithelial cell line derived from an NPC xenograft of Southern Chinese origin [30]. This C666-1 NPC cell line is the most representative NPC cell line to date, since it retains the native EBV while other NPC-derived cell lines have lost their EBV through the *in vitro* culture. The C666-1 genome was sequenced in paired-end 100 bases protocol on an Illumina HiSeq 2000 platform (Illumina, San Diego, CA, USA). With a total of 251 gb of output data, the EBV coverage reaches 504 folds despite the process not involving PCR amplification or other methods of enrichment. The consensus EBV sequence was constructed through reference mapping and Sanger sequencing [18]. The C666-1 EBV was found to be phylogenetically close to other Chinese NPC sequences GD1, -2, and HKNPC1.

More recently, two EBV genomes in immortalized human B lymphocyte cell lines were sequenced using the Illumina MiSeq platform [19]. These cell lines were derived from peripheral blood of two healthy donors. Sequencing reads from total DNA of the cell lines were mapped to the EBV reference genome and the mapped reads were assembled by CLC Genomic Workbench to give the two EBV genomes, K4413-Mi and K4123-Mi. In the same study, NA12878 EBV genome, which represents the EBV in peripheral blood of a Caucasian subject, was assembled using the data from the 1000 Genome Project [19]. Similarly, ten EBV sequences were constructed from the reads unmapped to human-generated from sequencing the lymphoblastic cell lines from the 1000 Genome Project [20]. These studies have demonstrated that published human genomic data may contain viral sequences useful in EBV studies.

Sequencing expense incurred by the presence of the much more abundant cellular genomic DNA inherent in standard DNA preparations can be prohibitive to large scale sequencing of viral genomes. These studies have shown that it is possible to assemble EBV as a side product of sequencing of cellular DNA, provided there are high copy numbers of EBV and a high throughput of sequencing data.

### 3.2. Next-Generation Sequencing with EBV Enrichment

#### 3.2.1. Enrichment by Induction of Lytic Viral Replication

Akata and Mutu are Burkitt’s lymphoma cell lines which are commonly-used model cell lines. The sequencing of EBV strains in Akata and Mutu cell line was made feasible by taking advantage of the properties of lytic replication of EBV in these lines to increase the viral copy number. Induction of viral lytic replication was performed by incubating the cells in medium containing anti-immunoglobulinA (anti-IgA) and anti-IgG [23]. Subsequently, the relative amount of cellular DNA was reduced by Hirt DNA extraction method. Their EBV genomes were determined by paired-end 100-base sequencing and were *de novo* assembled using reads aligned to EBV reference [23].

#### 3.2.2. Enrichment by PCR

Induction of lytic replication can only be applied to certain cell lines. One way to increase the abundance of viral DNA in non-inducible lines or primary specimens is by PCR amplification. EBV from a primary NPC biopsy, HKNPC1, was PCR-amplified using 60 primer pairs and the Illumina Genome Analyzer II platform was used to determine its sequence [25]. The HKNPC1 EBV genome was generated by mapping the reads to the wild type EBV and calling the consensus sequence from the alignment. As much as 90% of total usable reads were mapped to type 1 EBV reference, hence, signified how PCR enrichment can greatly increase cost-effectiveness in determination of the viral sequence. Similarly, the EBVs of spontaneous LCLs established from peripheral blood mononuclear cells (PBMC) of four Kenyan children were PCR-amplified by using 59 pairs of primer [26]. The pooled PCR products were sequenced by Illumina MiSeq platform, and there were more than 90% of reads in each sample could be aligned to reference EBV genome.

#### 3.2.3. Cloning into F-Factor Plasmids

The M81 EBV was cloned into the F-factor-based replicon in *E. coli* [31], at the EBV terminal repeats [24]. The genome of M81 was then determined by Illumina HiSeq 2000 sequencing and assembled by GS Reference Mapper software [24]. Standard Sanger sequencing was used to confirm ambiguous regions and repeat regions. The study showed that the M81 EBV exhibits a higher tissue tropism towards epithelial cells than B95-8 [24]. The virus sequence was also found to be highly similar to viruses isolated from Chinese NPCs. Polymorphisms in BZLF1 gene have been pointed by recombinant virus assays to have contributed to this phenomenon. It proved the value of genomic sequencing of EBV strains from different geographic localities and disease origin.

#### 3.2.4. Target DNA Capture by Hybridization

Amplicon sequencing of HKNPC1 has signified how target enrichment can significantly increase the proportion of viral DNA to host cellular DNA and, hence, greatly increase efficiency in utilizing the capacity of next-generation sequencing technology. However, the success in sequencing of HKNPC1 is not sufficient to drive projects of large-scale sequencing of EBV genomes. Amplicon sequencing works well for sequencing of single samples, but the procedures of PCR, following by purification and normalization of PCR products, can be time-consuming and labor-intensive for large numbers of samples. Moreover, PCR amplification can be a source of sequence errors, even if high-fidelity polymerases are used [32]. It is also difficult to control for the pooling of PCR products into equal molar concentration across the whole genome.

The technology of target DNA enrichment by hybridization can be traced back to traditional techniques for enriching nucleic acids with biotinylated DNA bait [33]. Modifications of this technique have been made to serve different purpose, for example, to detecting low-frequency variants and recombinant DNA molecules from a DNA pool [34]. Exome sequencing, which also known as targeted exome capture, utilized the enrichment-by-hybridization approach to enrich exonic DNA, which is approximately 1% of the entire human genome [35]. The same principle has been applied to capture the target DNA of interest, in our case, the EBV DNA.

##### RNA Bait

The SureSelect target enrichment system has been used to successfully enrich and sequence EBV and KSHV DNA [36]. RNA probes of 120 bp in length, overlapped at 5x, have been designed to cover the EBV genome. Three published EBV (B95-8, C666-1, and HKNPC1) genomes were first re-sequenced to validate the sequencing workflow. The whole sequences of eight NPC biopsy-derived EBV (NPC-EBV) genomes, designated HKNPC2 to -9, were then determined [27].

The same hybridization capture system has been utilized in a study to determine the genomic sequences of 71 geographically-distinct EBV strains from cell lines, multiple types of primary tumors such as NPC, Burkitt’s lymphoma and Hodgkin’s lymphoma, blood samples, and a saliva sample [28]. The study greatly enriched the genomic data of EBV strains, in particular those from East Africa and Australia. It is also the most comprehensive study on geographic distribution of strains defined on the level of whole EBV genome.

##### DNA Probe

Target capture through hybridization enabled the sequencing of EBV with greater time- and cost-effectiveness than PCR-based enrichment. The higher coverage uniformity also improved the quality of contigs from *de novo* assembly. However, the method still leaves us the task of joining the contigs to form complete genomes. The more fragmented the contigs are, the greater effort is needed to join them. With the aim of improving the capture system from sequence uniformity to contig quality, we utilized individually-synthesized DNA oligos as probes for EBV capture. We also increased the read length from paired-end 100 bp to 300 bp. The experimental detail is described in section five.

## 4. Construction of EBV Genomes Using NGS Data

### 4.1. Reference Mapping and Consensus Calling

Bioinformatics analysis of next-generation sequencing data is crucial to convert the raw output data to something of biological interest. Two major approaches are reference mapping and *de novo* assembly. With reference mapping each read is aligned to the reference genome. This is common in re-sequencing projects which the genomic sequence of the organism is already known and one is only interested in the variations among different strains of the same species. The commonest short-read alignment programs have been developed based on the Burrow-Wheeler transform (BWT) algorithm. Examples of programs using this algorithm include BOWTIE [37], SOAP2 [38] and the one used in this study, namely Burrow-Wheeler Aligner (BWA) [39]. This algorithm first creates an efficient index of the reference genome in order to facilitate rapid searching under limited system memory. The trade-off for this fast BWT method is the sensitivity of finding alignments. BWA is only able to find alignments within a certain “edit distance” to the reference genome sequence [39]. Edit distance can be described as the number of operations required to transform one sequence to another, which affected by the number of gaps and mismatch between the reads and the reference genome. As a result of this limitation of edit distance, large insertions which exceeded the size of the allowed mismatch will not be detected. Moreover, mismatches occur more frequently and polymorphic regions, such as EBNA-2, -3, and LMP-1 and -2, where sequence reads most probably differ from the reference, and hence, reduce the validity of the consensus sequence generated from reference mapping. Repeat regions are also prone to misalignment and very often result in a high number of reported variations in the consensus sequence. A great effort for validation is required in these regions of the EBV genome.

For data generated from sequencing protocol without EBV enrichment, it is encouraged to first align the reads to the human genome and use the unmapped reads for further EBV analyses. The EBV genome contains genes that are homologous to human genes. For example, BHRF1 protein from EBV is a homolog of human Bcl-2 [40], while BCLF1 is a homolog to human interleukin-10 [41]. Removing human reads reduces the possibility of mistaking human sequences as EBV sequences. It also greatly reduces the file size and the demand of computing power in downstream analyses.

With reference-mapping one assumes that the new genome sequenced is highly similar to the reference sequence. There are two NCBI reference EBV genomes, one for each major type of EBV: type 1 (Accession no. NC_007605.1) and type 2 (NC_009334.1). Mapping the reads to one of the references will normally suffice to identify the type of EBV sequenced, since gaps of no reads will be observed at type-specific genes EBNA-2 and -3 when data of type 1 EBV is mapped to the type 2 reference, and *vice versa*. However, it is recommended to map the reads to both EBV references since, in some uncommon scenarios, both types of the virus may co-exist in a specimen. Future publications should also report exactly which of the reference sequences to be used.

Consensus calling constructs EBV genomes using mapped data. The dominant nucleotide species from the reads mapped along the reference will be called at every single position and considered as the consensus sequence. The minor nucleotide species will be either be regarded as sequencing or mapping error, or inferred that a coinfection of minor viral strain is present. In the study of HKNPC1 amplicon sequencing, we defined a position to be homogeneous if the variant frequency is ≥95% and a position to be heterogeneous if the variant frequency is between 20% and 94% [25]. Multiple nucleotide species is very commonly observed in repeat regions due to misalignment of reads, therefore meticulous validation should be conducted by pyrosequencing or other techniques. Nucleotide positions with read depth less than five were classified as ambiguous sites as there is insufficient depth to make a high confidence call. Since these cut-offs are arbitrary and are subjected to adjustment in different sequencing protocols, it is advised to determine the error rate and percentage cut-off for genuine minor variants experimentally through manual mixing two different strains of the virus.

### 4.2. *De Novo* Assembly

*De novo* sequencing involves sequencing a novel genome without the aid of external data. It does not depend on existing reference genomes and, hence, can reveal large genetic variations or regions differing significantly from the references. The general prerequisites for high-quality assemblies include high base quality of reads and uniform coverage.

**Figure A1:**
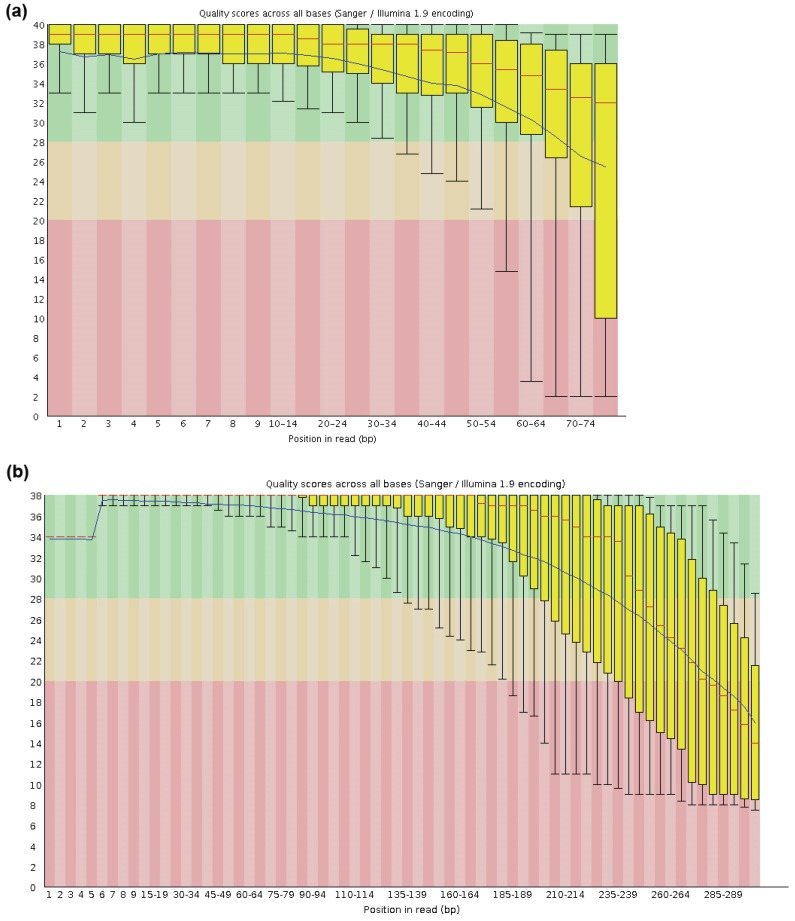
Per base sequence quality of capture sequencing of B95-8 EBV on Genome Analyzer IIx 76 bp protocol (**a**) and MiSeq 300 bp protocol (**b**). These quality profiles are representative of other samples in the same run.

In comparison to reference mapping, where sequence error is adjusted by cut-offs during the variants and consensus calling, it is necessary to ensure a good quality of input sequence data in *de novo* assembly. Each base in a read is assigned a quality score by the Illumina sequencing platform using a phred-like algorithm [42,43], the distribution of mean quality scores is a rough estimation of the quality of the sequencing run. The per-sequence quality scores module of the FastQC software reports the distribution of mean quality scores of individual reads and this serves as the basis for trimming. There are at least two main approaches in removing low quality bases from reads. The first is to select a single cut-off for all reads, based on a summary of base quality data. The choice of trimming length is a compromise between base quality and desired read length for assembly. In a typical 300 bp MiSeq run the lower quartile of quality score per base penetrated Q20 at approximately 230 bp from 5' end. Removing the last 70 bases from 3' end ensures only high quality bases in a read are retained. In a paired-end 76 bp run where the overall base quality is generally higher, a more stringent trimming regime might be tolerated and only the last six bases are trimmed (Figure A1).

The second main approach is to trim base on the sequence quality for individual reads. The length of reads after trimming varies because of the variable base quality in each reads. A number of software is available for this purpose and is reviewed by del Fabbro and colleagues [44]. The best algorithm is sample-dependent and requires trial-and-error.

**Figure 1:**
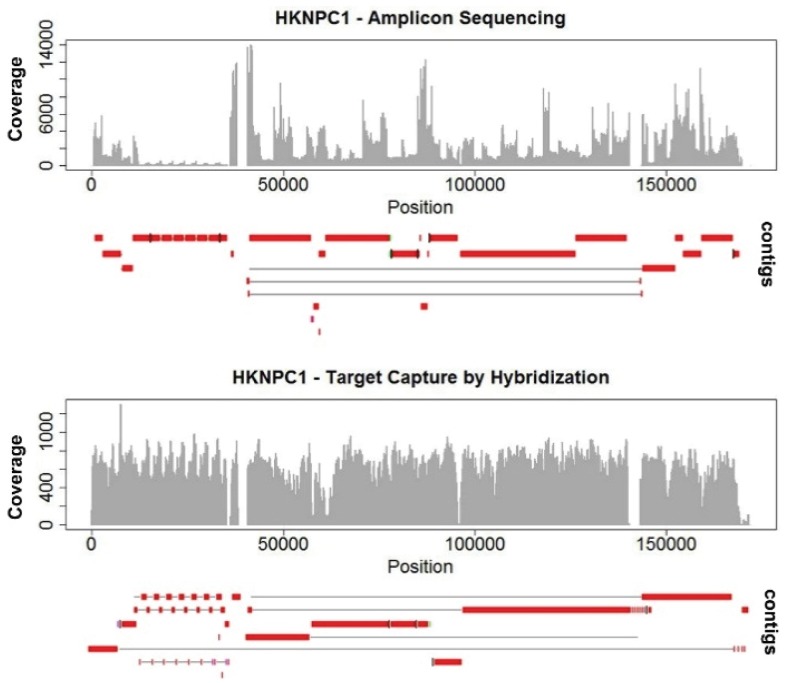
Coverage and alignment of contigs of HKNPC1 EBV by amplicon sequencing and target capture. Uneven read coverage is observed in amplicon sequencing of HKNPC1. More uniform coverage is observed in HKNPC1 enriched by target capture through hybridization using Agilent RNA-bait. The contigs assembled in amplicon sequencing are more fragmented than that from target capture.

Algorithms of most genome assembly software make use of information on expected coverage to infer genome assemblies and copy number variations. It directly affects the quality of *de novo* assemblies. The highly-uneven coverage in HKNPC1 obtained by the amplicon sequencing approach resulted in more fragmented contigs, with 25 contigs alignable to the reference EBV, compared with 15 contigs from the data of target capture sequencing (Figure 1). Uneven coverage poses problems mainly in repeats. The amplicon sequenced HKNPC1 failed to be assembled into contiguous sequence in almost all major and minor repeats of EBV. Read uniformity is particularly important at low sequencing depth. This is often the case for sequencing samples of low EBV viral load.

The assembler used in the study of sequencing HKNPC2 to -9 [32], namely Velvet [45], was developed based on the de Bruijn graphs method to resolve the assembly of high depth and short read data generated from NGS technology. This algorithm was originally developed for problems in combinatorial mathematics [46]. It is based on graphs of small, fixed-length subsequences know as k-mer, where k equals to the subsequence length. The assembly result strongly depends on this value of k and other parameters, such as expected average k-mer coverage (exp_cov) and the lowest coverage of nodes to be excluded (cov_cutoff). These parameters, particularly the value of k, are difficult to estimate without running through different values of these parameters and scrutinize the result manually. Case-by-case optimization is required even for assembling genomes of the same species and data from the same sequencing protocol.

**Figure 2:**
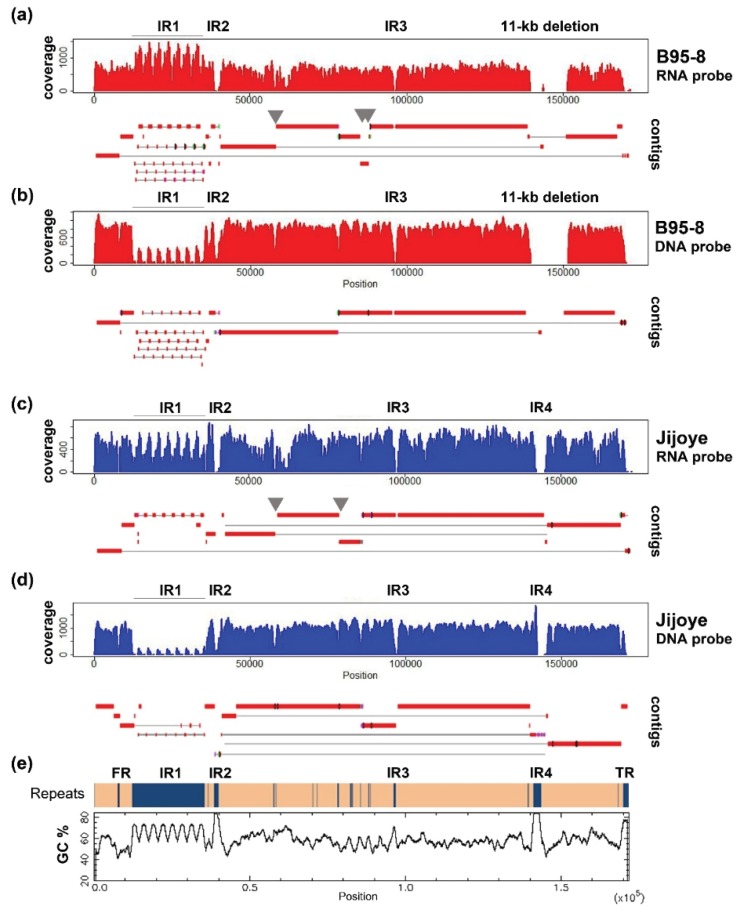
Coverage and alignment of contigs of B95-8 and Jijoye EBV by different capture strategies and sequencing protocols. (**a**) B95-8 EBV captured by RNA bait and sequenced by MiSeq PE150; (**b**) B95-8 EBV captured by DNA probe and sequenced by MiSeq PE300; (**c**) Jijoye EBV captured by RNA-bait and sequenced by MiSeq PE150; (**d**) Jijoye EBV captured by DNA probe and sequenced by MiSeq PE300; and (**e**) distribution of repeat regions and GC percentage across the EBV genome. The GC percentage plot is created by EMBOSS Cpgplot.

Sanger sequence remains, to date, to be the gold standard for bridging the contigs and validation of variations. With our current target capture platform, contigs of most of the samples do share the similar positions of breaks at repeat regions (Figure 2e) of the EBV genome. Therefore. the same set of primers could be applied to bridge the gaps in most of the samples. This will greatly increase the efficiency of constructing complete EBV genomes when the study is scaled up to include hundreds of samples. With this aim of reducing contig gaps in mind, we tested another hybridization system for EBV capture.

## 5. Resequencing of Prototype Type 1 (B95-8) and Type 2 (Jijoye) EBV—A Comparison of DNA and RNA Probes

### 5.1. Cell Lines

B95-8 has been re-sequenced in multiple studies and, hence, served as a gold standard for sequence accuracy. Jijoye is re-sequenced in this study to validate the capability of the system to capture type 2 EBV.

### 5.2. DNA Probes

Customized Integrated DNA Technology (IDT) xGen^®^ Lockdown^®^ probes are 120 bp DNA oligos designed across the whole genome of type 1 EBV and selected regions of type 2 EBV. These oligos covered the genome at end-to-end (1x) coverage. The oligo pools are re-suspended and mixed according to the manufacturer’s protocol.

### 5.3. Library Preparation, Target Capture, and Sequencing Analysis

The MiSeq platform was used to analyze the EBV genomes in B95.8 and Jijoye cell lines. NEBNext^®^ Ultra™ DNA Library Prep Kit for Illumina^®^ (New England Biolabs, Ipswich, MA, USA) and SeqCap^®^ EZ Hybridization and Wash Kits (Roche Nimblegen, Madison, WI, USA) were used for library preparation and target capture. DNA oligo probes were synthesized as a pool of custom oligos by Integrated DNA Technologies (IDT). All library preparation, hybridization, and post-reaction clean-up steps were performed according to the Rapid Protocol for DNA Probe Hybridization and Target Capture Using an Illumina TruSeq (Version 2.0) observing all recommended quality control steps. Denatured DNA libraries were mixed with a PhiX control library. Cluster generation and 300-bp pair-ended sequencing were performed in succession using the MiSeq platform, according to manufacturer’s protocol.

### 5.4. *De Novo* Assembly of EBV Genomes

Sequencing reads from MiSeq Personal Sequencer were demultiplexed into individual samples by allowing one mismatch in the index sequence. Quality assessment and filtering on the raw reads were carried out to remove reads containing adaptor sequences. Coverage of reads was assessed by mapping untrimmed reads of each sample to the type 1 (NC_007605) and type 2 (DQ279927) reference EBV genomes by BWA software. Bases of poor quality at the end of sequence reads were trimmed. These reads were assembled using a de Bruijn graph assembler Velvet [45]. Location and orientation of contigs were evaluated by pairwise aligning of the contigs to the reference EBV genomes using NCBI alignment tools.

### 5.5. Results

EBV DNA libraries captured by DNA probes and sequenced in pair-ended 300 bp run were trimmed to 230 bp and utilized in assembling the contigs. Using a k-mer length of 55, an expected k-mer coverage of 650, and a k-mer coverage cut-off of 50, the output graph for B95-8 has 94 nodes. The maximum contig size is 38,247 bp and N50 equals 42,581. Using a k-length of 45, an expected k-mer coverage of 900, and a k-coverage cut-off of 200, the output graph for B95-8 has 91 nodes. The maximum contig size is 42,247 bp and N50 equals 39,849 bp. The coverage profile and alignment of contigs to the reference sequences are illustrated in Figure 2.

We compare the data to that produced from different reagents and sequencing protocols, in which EBV DNA libraries were captured by RNA bait and sequenced in pair-ended 150 bp run. The method was described in details in a previous publication [27]. The output reads were trimmed to 100 bp, and then utilized in assembling the contigs. Using a k-mer length of 37 and a k-mer coverage cut-off of 50, the output graph for B95-8 has 70 nodes. The maximum contig size is 50,083 bp and N50 equals 17,546. Using a k-mer length of 37, an expected k-mer coverage of 400, and a k-coverage cut-off of 50, the output graph for Jijoye has 45 nodes. The maximum contig size is 44,262 bp and N50 equals 19,989 bp.

Most of the non-repeats regions are assembled reasonably well in both approaches. Increasing the read length to paired-end 300 bp helped significantly to resolve minor repeats. Repeats in BPLF1 (NC_007605 coordinates 57,396–57,642; 58,099–58,233), EBNA3A (81,920–82,781), and -3B (85,234–85,410) genes (grey arrows in Figure 2), which were broken in contigs in 150 bp run, were bridged in contigs of the 300 bp run. However, increasing read length does not help to close the gaps at internal repeats 1, -2, -3, and -4, and repeats at the OriP region, since they either have a large repeating unit, as in internal repeat 1 (IR1), or the entire repeat length is too large to be covered in a single read (e.g., IR4). Some of these difficult regions are of particularly high GC content (over 80% in IR2 and -4, shown in Figure 2e) which poses difficulties in generating high-quality reads and assembly.

## 6. Future Development

One of the bioinformatics strategies used to improve the quality of assemblies is by utilizing variable sizes of k-mer to building of de Bruijn graphs, and merges the results from different inputs of k. This was suggested to improve quality and length of contigs in transcriptome analysis [47] and metagenomic analysis [48]. Application of this strategy to EBV genome assembly might further improve contig quality by bridging some of the gaps due to minor repeats.

The major repeats, including internal repeats (IR), terminal repeats (TR), and family of repeats (FR) in the OriP region remain to be an obstacle for completing the EBV genome. Single molecule real-time sequencing (SMRT) technology is able to sequence ultra-long reads of on average 10,000 bp or longer. The platform has been applied to sequence microbial genome and was able to obtain almost gapless contigs upon assembly [49]. Not being vulnerable to GC bias, it can potentially sequence through difficult regions such as IR2 and -4. When target capture and sample preparation for SMRT sequencing is better established and the cost become less prohibitive, it might greatly facilitate construction of EBV genomes in large scale sequencing projects.

With growing number of EBV genomes available, it becomes increasing important to design studies that can reveal biological significance of the genomic variations of the virus. Consideration of disease associations, for example, must include analyses of appropriate control sequences. EBV that persists naturally in immunocompetent hosts without noticeable symptoms from the same geographic region and ethnic group serve as useful controls since it minimizes the effect of geographical variations among EBV genomes. Although little is known about the correlation of age and sex with viral strains, it is of common practice to include age- and sex-matched control to ensure only the condition of interest is interrogated. Since EBV is known to be tropic to certain cell types, in particular B-cells and epithelial cells, specimens from different body compartments (peripheral blood mononuclear cells, plasma, saliva, nasopharyngeal biopsies, *etc.*) of the same individual, or viruses from the same compartment of different individuals, will shed light on the cell-type-specific effect of the virus.

This review provides an overview of the development of EBV genomic sequencing since the first EBV genome obtained. It also described recent advances and potential future directions for EBV sequencing projects. On the basis of these advances, future work of EBV genomic sequencing will facilitate a better understanding of the genetic variations of EBV and discovery of potentially-pathogenic variants.

## Figures and Tables

**Table 1:** Summary of sequencing methods of published EBV genomes.

Method	Genomes	Accession No.	NGS Read Length *	Ethnicity	Tissue/Fluid of Origin *	Cultured Cell Type *	Bioinformatics Strategy	Software Used	Reference
Shotgun sequencing	B95-8	V01555	n/a	N. American	IM	LCL	n/a	n/a	[11]
	AG876	DQ279927	n/a	African	BL	BL	n/a	n/a	[16]
	GD1	AY961628	n/a	Chinese	NPC saliva	LCL	n/a	n/a	[15]
NGS without EBV enrichment	GD2	HQ020558	PE44	Chinese	NPC biopsy	not applicable	Reference mapping	SOAPdenovo	[17]
	C666-1	KC617875	PE100	Chinese	NPC	mouse xenograft	Reference mapping	BWA, GATK, Samtools	[18]
	K4413-Mi	KC440851	PE175	N. American	PBMC	spLCL	*De novo* assembly	CLC Genomic Workbench	[19]
	K4123-Mi	KC440852		N. American		spLCL			
	NA12878	n/a	PE36	N. American		LCL			
	NA20783	n/a	n/a	European	PBMC	LCL	Reference mapping	BWA, Samtools	[20]
	NA18507			African					
	NA20348			African					
	NA18923			African					
	NA20524			African					
	NA19114			European					
	NA19474			African					
	NA19315			African					
	NA19380			African					
	NA19384			African					
	GC1	KP735248	PE100	Korean	GC	GC	Reference mapping	BWA, Samtools	[21]
	CCH	KP968257	PE250	S. American	BL	not applicable	Reference mapping	CLC Genomic Workbench	[22]
	MP	KP968258		S. American					
	SCL	KP968259		S. American					
	VGO	KP968260		S. American					
	RPF	KR063344		S. American					
	FNR	KR063345		S. American					
	CV-ARG	KR063343		S. American					
	HU11393	KP968261		African					
	H03753A	KR063342		African					
	H018436D	KP968262		African					
	H058015C	KP968263		African					
	H002213	KP968264		African					
EBV enrichment by lytic induction	Akata	KC207813	PE100	Japanese	BL	BL	*De novo* assembly	Velvet	[23]
	Mutu	KC207814		African					
F-factor cloning	M81	KF373730	n/a	Chinese	NPC	LCL	n/a	GS Reference Mapper	[24]
Amplicon sequencing	HKNPC1	JQ009376	PE76	Chinese	NPC biopsy	not applicable	Reference mapping	BWA, Samtools	[25]
	LCL1	n/a	PE150	African	PBMC	spLCL	Reference mapping	Strand NGS	[26]
	LCL3								
	LCL9								
	LCL10								
EBV capture by hybridization	HKNPC2	KF992564	PE76	Chinese	NPC biopsies	not applicable	*De novo* assembly	Velvet	[27]
	HKNPC3	KF992565							
	HKNPC4	KF992566							
	HKNPC5	KF992567							
	HKNPC6	KF992568							
	HKNPC7	KF992569							
	HKNPC8	KF992570							
	HKNPC9	KF992571							
	71 EBV genomes	See ref.	PE76	Mixed	NPC biopsy,	Mixed	*De novo* assembly	Velvet	[28]
					Healthy saliva,				
					HL, BL,				
					PTLD & IM				
	EBVaGC1	KT273942	PE125	Chinese	EBVaGC	not applicable	*De novo* assembly	Velvet	[29]
	EBVaGC2	KT273943							
	EBVaGC3	KT254013							
	EBVaGC4	KT273944							
	EBVaGC5	KT273945							
	EBVaGC6	KT273946							
	EBVaGC7	KT273947							
	EBVaGC8	KT273948							
	EBVaGC9	KT273949							

* PE: Paired-end, followed by the length of reads in base-pair; spLCL: spontaneous lymphoblastoid cell line; LCL: lymphoblastoid cell line infected with external virus source; BL: Burkitt’s lymphoma; PTLD: post-transplant lymphoproliferative disease; PBMC: peripheral blood mononuclear cells; GC: gastric carcinoma; IM: infectious mononucleosis; EBVaGC: EBV-associated gastric carcinoma; n/a: information not available.
